# Coffee and cigarettes: Examining the association between caffeinated beverage consumption and smoking behaviour among youth in the COMPASS study

**DOI:** 10.1016/j.pmedr.2020.101148

**Published:** 2020-06-18

**Authors:** Matthew James Fagan, Katie Mary Di Sebastiano, Wei Qian, Scott Leatherdale, Guy Faulkner

**Affiliations:** a Population Physical Activity Laboratory, School of Kinesiology, University of British Columbia, Vancouver, BC, Canada; b School of Public Health and Health Systems, University of Waterloo, Waterloo, ON, Canada

**Keywords:** Smoking, Adolescent, Caffeine, Energy drinks, Sugar sweetened beverages, E-cigarettes, Vaping

## Abstract

In adults, coffee, sugar-sweetened beverage (SSB) and high energy drink consumption have been related to increases in risky behaviour, including smoking. However, these associations are not well understood during adolescence. The purpose of this study was to examine the association between beverage consumption and smoking behaviour among Canadian adolescents. Using data from the COMPASS study (2016–2017; n = 46,957), four models were developed to investigate whether beverage consumption explained variability in smoking behaviour in adolescence (age = 15.7 ± 1.2 yrs); 1) smoking status; 2) e-cigarette use status; 3) days smoking cigarettes per month; and 4) days using an e-cigarette per month. Models were adjusted for demographic factors. Logistic (models 1 and 2) and ordinal logistic (models 3 and 4) were used for analysis. An association between the frequency of SSBs, coffee/tea or high energy drinks consumption and smoking behaviour was identified in all models. Greater beverage consumption was associated with being a current smoker (OR = 2.46 (2.02, 2.99)), former smoker, (OR = 2.50 (1.53, 4.08)), and currently using an e-cigarette (OR = 4.66 (3.40, 6.40)). Higher beverage consumption was also associated with more days smoking/using an e-cigarette per month (OR = 2.67 (1.92, 3.70) and 3.45 (2.32, 5.12), respectively). High energy drink consumption on 4 or 5 days of the school week was the best predictor of smoking behaviour in all models. Given the health consequences of smoking and e-cigarette use and their association with SSB, high energy drinks and coffee consumption, policy initiatives to prevent smoking initiation and limit access to these beverages needs ongoing attention and implementation.

## Highlights

• Caffeinated beverage consumption is associated with smoking behaviours in youth.
• High energy drink consumption is most strongly associated with smoking behaviours.
• Caffeinated beverages and smoking behaviour demonstrate a dose–response association.

## 1 Introduction

Tobacco use remains a leading risk factor contributing to the burden of disease in Canada (Alam et al., 2019). Approximately 15% of the Canadian population are considered cigarette smokers, including 10.6% of Canadian youth (aged 15–19) (Reid et al., 2019). Further, 15% of Canadians have used an e-cigarette, with adolescents and young adults reporting the highest rate of e-cigarette use of any age category, with 23% of students in grades 7–12 having ever tried an e-cigarette (Reid et al., 2019). Since the introduction of e-cigarettes, the prevalence of their use has continued to rise in the youth population. This is concerning as e-cigarette use is a risk factor for subsequent cigarette smoking (Greenhill et al., 2016, Soneji et al., 2017). Further, evidence suggests that smoking in adolescence decreases the likelihood of quitting smoking in adulthood (Chen and Millar, 1998). Continued attention to preventing smoking initiation during adolescence should be a public health priority.

Adolescents are exposed to a wider variety of beverage choices compared to children, and as autonomy increases, so too does experimentation with beverages, such as with those containing caffeine. For example, 73% of US youth consume caffeine daily, with the majority of their caffeine consumption coming from sugar-sweetened beverages (SSB), high energy drinks and coffee (Branum et al., 2014). Increased SSB consumption, in particular, is associated with a range of negative health outcomes such as Type II diabetes, cardiovascular disease and obesity (Malik et al., 2010).

Notably, coffee consumption is associated with smoking behaviour in adults (Swanson et al., 1994). Heavy coffee drinking may predict increased smoking behaviour in the adult population, which is attributed to its caffeine content (Istvan and Matarazzo, 1984). A dose–response relationship has also been demonstrated between coffee consumption and smoking (Klesges et al., 1994). Additionally, caffeinated energy drinks have been related to substance use, including smoking in both middle school (Age: 11–13) (Mann et al., 2016) and high school students (Age 14–18) (Kearns et al., 2018). A longitudinal study of Finnish adolescents (age: 12–13 at time one and 15–16 at time two) found that high energy drink consumption predicted e-cigarette use and smoking (Kinnunen et al., 2018). However, SSBs are consumed in higher quantities than both coffee and high energy drinks by youth (Branum et al., 2014), and therefore may represent a significant source of caffeine in this population. Caffeinated energy drinks are considered a subclass of SSBs, as they are high in both caffeine and sugar. However, not all SSBs contain caffeine, and many of the standard measures used to capture SSB consumption do not differentiate beverages on caffeine content (Riordan et al., 2017). Regardless of caffeine content, SSBs have demonstrated the same positive association between consumption and smoking as coffee in adults (Kearns et al., 2018, Kristal et al., 2015). As youth demonstrate different caffeinated beverage consumption patterns as compared to adults, identifying which caffeinated beverage source has the strongest association with smoking behaviour may help to inform health policy for this population.

This exploratory study aimed to examine the association between caffeine consumption through various means and smoking behaviour in Canadian youth. We specifically aim to examine the association between beverage consumption and e-cigarette use and to determine which beverages are most strongly related to e-cigarette use as these associations remain relatively unexplored in youth. Our hypotheses included; increased consumption of caffeinated drinks will be associated with increased smoking behaviour (current smoker, current e-cigarette user, number of days smoking per month and times using e-cigarettes per month) in students and increased consumption of SSB will be associated with increased smoking behaviour in students independent of age, sex, BMI, school clustering or ethnic background of the students.

## 2 Methods

### 2.1 Data

This study uses data from the COMPASS study, an ongoing cohort study (2012–2021) collecting hierarchical and linked longitudinal behavioural and program/policy data in Canadian youth. The entire cohort sample (2016/2017) includes 46,957 grade 9–12 students (age: 13–17) at 95 secondary schools in Ontario (n = 34078/68), Alberta (n = 2982/9), British Columbia (n = 3617/5), Quebec (n = 6185/11) and Nunavut (95/2). The sample size for the individual variables reflects missing survey data as reported in Table 1. A full description of the COMPASS study methods is available in print (Leatherdale et al., 2014) and online (www.compass.uwaterloo.ca). The study was reviewed by the University of Waterloo Office of Research Ethics and appropriate school board committees.

**Table 1:** Demographic and descriptive statistics of participants.

		N (%)	Mean (sd)
Grade	9	10,537 (26.3)	
	10	11,136 (27.8)	
	11	9985 (24.9)	
	12	8432 (21.0)	
Age (years)			15.7 (1.2)
	13	395 (1.0)	
	14	7657 (19.1)	
	15	10,700 (26.7)	
	16	10,121 (25.2)	
	17	8392 (20.9)	
	18	2830 (7.1)	
Sex	Female	19,575 (49.3)	
	Male	20,142 (50.7)	
Race	White	27,724 (68.8)	
	Black	1634 (4.1)	
	Asian	2857 (7.1)	
	Hispanic	1025 (2.6)	
	Other/mixed	5509 (13.7)	
BMI			22.3 (4.4)
	Underweight	624 (2.1)	
	Healthy Weight	21,719 (71.3)	
	Overweight	5445 (17.9)	
	Obese	2666 (8.8)	
School median income ($)	25001–50000	3316 (8.2)	
	50001–75000	20,200 (50.2)	
	75001–100000	13,992 (34.7)	
	>100000	2764 (6.9)	
Never users of cigarettes		37,280 (93.0)	
Current user of cigarettes		2510 (6.3)	
Former user of cigarettes		306 (0.8)	
Current user of e-cigarettes		4647 (11.4)	
Non-user of e-cigarettes		36,125 (88.6)	
Days per school week drinking SSB			1.6 (1.6)
Days per school week drinking high energy drinks			0.3 (0.9)
Days per school week drinking coffee/tea with sugar			1.4 (1.7)
Days per school week drinking coffee/tea without sugar			0.6 (1.4)

*Note.* BMI = body mass index. SD = standard deviation.

### 2.2 Student level recruitment and participants

COMPASS schools provided permission to use active information passive-consent parental permission protocols, wherein the parent(s) or guardian(s) of eligible students are mailed an information letter and asked to contact the recruitment coordinator should they wish to withdraw their child from participation. All students not withdrawn were deemed eligible to participate, although a student could decline to participate at any time.

### 2.3 Data collection tools

All data used in the current study, except for school median income, was collected using the student-level questionnaire for COMPASS (Cq). School median income was determined through the median income for the forward sortation area (sortation area was determined by the first three alphanumeric digits of the postal code of the school). The questionnaire collects individual student data pertaining to multiple behavioural domains, correlates of the behaviours, and demographic characteristics. In each school, the Cq was used to collect within-school samples during class time. The Cq items are based on the national standard or current national public health guidelines (Leatherdale et al., 2014).

### 2.4 Measures

#### 2.4.1 Smoking behaviours

Four smoking behaviour related outcome variables were identified: current smoker, current e-cigarette user, days smoking cigarettes in the last 30 days, and times used an e-cigarette in the last 30 days. Current smokers were identified through two questions: “Have you ever smoked 100 or more whole cigarettes in your life?” and “On how many of the last 30 days did you smoke one or more cigarettes?”. Current smokers were identified through the report of smoking >100 cigarettes and any smoking in the previous 30 days. Similarly, current e-cigarette users were identified by the question: “Have you ever tried an electronic cigarette, also known as an e-cigarette?” and “On how many of the last 30 days have you used an e-cigarette?” This method of assessing smoking status is the only validated measure (Wong et al., 2012) for determining smoking status in the COMPASS study. With this method, 3 categories of smoking status for combustible cigarettes are created: current, former and non-smokers. However, due to the nature of the questions, it is only possible to create 2 e-cigarette usage categories: e-cigarette users and non-e-cigarette users. As such, these validated smoking variables will be used for all subsequent analyses.

#### 2.4.2 Beverage consumption behaviour

Four beverage consumption behaviours were identified: frequency of SSB consumption, frequency of high energy drinks consumption, frequency of coffee and tea with sugar consumption, frequency of coffee and tea without sugar consumption. All beverage consumption questions were part of a larger question about eating habits and therefore structured the same way. The question stated: In a usual school week (Monday to Friday), on how many days do you do the following? Drink sugar-sweetened beverages (soda pop, Kool-Aid, Gatorade, etc.) Do not include diet/sugar-free drinks; Drink high energy drinks (Red Bull, Monster, Rock Star, etc.); Drink coffee or tea with sugar (include cappuccino, Frappuccino, iced-tea, iced-coffees, etc.); and Drink coffee or tea without sugar. Response options included: None, 1 day, 2 days, 3 days, 4 days or 5 days.

### 2.5 Covariates

The following variables were controlled for in all models as they may be related to smoking behaviour or beverage consumption: grade (9, 10, 11, 12), ethnicity (white, black, Indigenous, Asian, Hispanic/Latin), Age (13, 14, 15, 16, 17, 18), sex (female, male), school area median income, and body mass index (BMI) calculated from self-reported height (cm) and weight (kg).

### 2.6 Statistical analysis

Descriptive statistics were used to assess differences in demographics and beverage consumption between students who engage in smoking behaviours and students who do not (Student’s *t*-test for continuous variables and chi-square test for categorical variables). If variables were skewed, the Wilcoxon rank-sum test was used. All analyses were run using SAS software package 9.4 (Cary, NC).

Four separate models were developed for 1) current/former smokers; 2) current e-cigarette users; 3) days smoked cigarettes per month, and 4) days used e-cigarette per month. All models were adjusted for age, sex, BMI, school median income, ethnicity and school clustering. Models 1 and 2 used multiple logistic regression and model 3 and 4 used ordinal logistic regression to investigate the ability of beverage consumption to explain the variability in smoking behaviour. The significance level was set at 0.05 (two-sided test). Each beverage type was tested individually in models. For all beverage variables, 0 was the referent. In smoking models, the non-smoker group was the referent. In e-cigarette models, the non-e-cigarette user group was the referent.

## 3 Results

### 3.1 Demographic and descriptive statistics

The majority of participants identified as male (50.7%), white (68.8%), and classified in the healthy weight BMI category (71.3%). Full demographic characteristics are shown in Table 1. SSB and coffee and tea with sugar were the most frequently consumed beverage (1.6 ± 1.6 days/week and 1.4 ± 1.7 days/week, respectively). More participants were classified as current e-cigarette users compared to current cigarette users (11.4% vs 6.3%). Additional descriptive statistics (Table 1) and a correlation matrix (Table 2) for primary outcome variables are included to describe the population and data set accordingly.

**Table 2:** Pearson Correlation Coefficient Matrix for Key Variables.

	Age	SSB	High-energy drinks	Coffee/Tea with Sugar	Coffee/Tea without Sugar	BMI	Cigarettes/day
Age p-value (n)	1.00000 40,555	−0.02683 <0.0001 39,379	0.02375 <0.0001 39,399	0.11299 <0.0001 39,422	0.10433 <0.0001 39,561	0.15076 <0.0001 30,952	0.10131 <0.0001 39,999
SSB p-value (n)		1.00000 39,539	0.24983 <0.0001 39,174	0.16346 <0.0001 39,188	−0.06470 <0.0001 39,313	0.00022 0.9695 30,284	0.09946 <0.0001 39,285
High-energy drinks p-value (n)			1.00000 39,560	0.22850 <0.0001 39,315	0.15430 <0.0001 39,383	0.05640 <0.0001 30,280	0.31827 <0.0001 39,311
Coffee/Tea with Sugar p-value (n)				1.00000 39,315	0.08613 <0.0001 39,389	0.03165 <0.0001 30,309	0.14325 <0.0001 39,334
Coffee/Tea without Sugar p-value (n)					1.00000 39,723	0.03401 <0.0001 30,416	0.06181 <0.001 39,481
BMI p-value (n)						1.00000 30,990	0.07103 <0.0001 30,685
Cigarettes/day p-value (n)							1.00000 40,173

### 3.2 Regression analysis

The relationships between smoking behaviour and beverage consumption was generally consistent across all models; therefore, general model interpretations are presented in text.

Frequency of SSB, coffee/tea or high energy drink consumption was associated with the likelihood of smoking behaviour (current smoker, former smoker, current e-cigarette user) and the frequency of smoking behaviour (more cigarettes smoked, and e-cigarette use per month). Generally, there was a dose–response association in which the more days of the week the students engaged in drinking any of these beverages, the greater the frequency of smoking behaviour with the exception of coffee/tea without sugar. The association was highest in all models when high energy drinks were used as the predicting variable. The largest association was found between high energy drink consumption on 5 days of the week and current e-cigarette use (OR = 3.45 (2.32, 5.15). Conversely, moderate beverage consumption (1–2 days per week) was associated with lower levels of smoking behaviour. For example, consuming SSBs, energy drinks and coffee with sugar on 1 day, had a smaller association with current smoking (OR = 0.71 (0.63, 0.80), 0.79 (0.69, 0.90), and 0.65 (0.57, 0.75)). Table 3, Table 4, Table 5, Table 6 present complete regression analysis results for Model 1, 2, 3 and 4, respectively.

**Table 3:** A logistic regression analysis of the association between beverage consumption and current/former smoking status among grade 9 to 12 students in 2016/2017 of the COMPASS study.

	Number of days consuming	P-value	Current smokersOR (CI)	p-value	Former smokers OR (CI)
Days per school week drinking sugar-sweetened beverages					
	0 (ref)				
	1	< 0.0001*	0.71 (0.63, 0.80)	0.0259*	0.70 (0.51, 0.96)
	2	0.8475	0.99 (0.88, 1.11)	0.6112	0.92 (0.68, 1.25)
	3	0.1805	1.09 (0.96, 1.24)	0.1196	1.28 (0.94, 1.76)
	4	0.3537	1.09 (0.91, 1.31)	0.6379	1.12 (0.70, 1.80)
	5	< 0.0001*	1.80 (1.60, 2.02)	0.0251*	1.45 (1.05, 2.01)
Days per school week drinking high energy drinks					
	0 (ref)				
	1	0.0006*	0.79 (0.69, 0.90)	0.2769	0.82 (0.56, 1.18)
	2	0.8931	1.01 (0.85, 1.21)	0.6332	0.88 (0.53, 1.47)
	3	0.0070*	1.34 (1.08, 1.65)	0.7352	1.11 (0.61, 2.00)
	4	< 0.0001*	2.09 (1.62, 2.71)	0.1037	1.81 (0.89, 3.68)
	5	< 0.0001*	2.46 (2.02, 2.99)	0.0003	2.50 (1.53, 4.08)
Days per school week drinking coffee/tea with sugar					
	0 (ref)				
	1	< 0.0001*	0.65 (0.57, 0.75)	0.0110*	0.60 (0.40, 0.89)
	2	0.9335	0.99 (0.88, 1.13)	0.0211*	0.61 (0.40, 0.93)
	3	0.0630	1.14 (0.99, 1.30)	0.7353	0.93 (0.63, 1.39)
	4	< 0.0001*	1.46 (1.24, 1.71)	0.0001*	2.07 (1.43, 3.00)
	5	< 0.0001*	1.96 (1.77, 2.18	<0.0001*	2.20 (1.68, 2.87)
Days per school week drinking coffee/tea without sugar					
	0 (ref)				
	1	0.9716	1.00 (0.83, 1.20)	0.8514	1.04 (0.68, 1.59)
	2	0.7080	0.96 (0.77, 1.20)	0.1387	0.63 (0.34, 1.16)
	3	0.0033*	0.65 (0.49, 0.87)	0.7874	0.93 (0.53, 1.62)
	4	0.8844	1.02 (0.75, 1.40)	0.1048	1.62 (0.90, 2.92)
	5	<0.0001*	1.65 (1.39, 1.96)	0.0476*	1.50 (1.00, 2.23)

Note. * indicates significance at p < 0.05. Models were adjusted for age, sex, race, body mass index category, and income group. Reference group for dependent variable = non-smokers.

**Table 4:** A logistic regression analysis of the association between beverage consumption and e-cigarette status use among grade 9 to 12 students in 2016/2017 of the COMPASS study.

	Number of days consuming a beverage	P-value	(CI)
Days per school week drinking sugar-sweetened beverages			
	0 (ref)		
	1	< 0.0001*	1.29 (1.17, 1.41)
	2	< 0.0001*	1.14 (1.26, 1.58)
	3	< 0.0001*	1.68 (1.51, 1.86)
	4	< 0.0001*	1.53 (1.32, 1.78)
	5	< 0.0001*	1.83 (1.64, 2.05)
Days per school week drinking high energy drinks			
	0 (ref)		
	1	< 0.0001*	2.50 (2.22, 2.81)
	2	< 0.0001*	3.05 (2.61, 3.56)
	3	< 0.0001*	3.57 (2.82, 4.52)
	4	< 0.0001*	4.66 (3.40, 6.40)
	5	< 0.0001*	4.06 (3.32, 4.96)
Days per school week drinking coffee/tea with sugar			
	0 (ref)		
	1	< 0.0001*	1.36 (1.23, 1.51)
	2	< 0.0001*	1.73 (1.56, 1.93)
	3	< 0.0001*	2.11 (1.90, 2.35)
	4	< 0.0001*	2.05 (1.74, 2.41)
	5	< 0.0001*	2.16, (1.95, 2.38)
Days per school week drinking coffee/tea without sugar			
	0 (ref)		
	1	0.3269	1.07 (0.94, 1.21)
	2	0.4868	1.08 (0.87, 1.33)
	3	0.5909	1.06 (0.86, 1.30)
	4	< 0.0001*	1.53 (1.25, 1.88)
	5	< 0.0001*	1.36 (1.18, 1.57)

Note. * indicates significance at p < 0.05. Models were adjusted for age, sex, race, body mass index category, and income group. Reference group for dependent variable = non e-cigarette users.

**Table 5:** An ordinal logistic regression analysis of the association between beverage consumption and number of days using cigarettes in the last 30 days among grade 9 to 12 students classified as smokers in 2016/2017 of the COMPASS study.

	Number of days consuming	P-value	OR (CI)
Days per school week drinking sugar-sweetened beverages			
	0 (ref)		
	1	0.0602	0.74 (0.54, 1.01)
	2	0.0343*	0.72 (0.53, 0.98)
	3	0.0072*	0.67 (0.50, 0.90)
	4	0.1580	0.78 (0.55, 1.10
	5	0.2857	1.19 (0.87, 1.62)
Days per school week drinking high energy drinks			
	0 (ref)		
	1	0.8897	0.98 (0.79, 1.23)
	2	0.5217	1.09 (0.84, 1.43)
	3	0.2358	0.82 (0.59, 1.14)
	4	0.2706	1.30 (0.82, 2.07)
	5	< 0.0001*	2.67 (1.92, 3.70)
Days per school week drinking coffee/tea with sugar			
	0 (ref)		
	1	0.5416	0.92 (0.70, 1.21)
	2	0.0154*	0.71 (0.54, 0.94)
	3	0.4473	0.91 (0.71, 1.17)
	4	0.5239	1.12 (0.79, 1.57)
	5	0.0772	1.25 (0.98, 1.59)
Days per school week drinking coffee/tea without sugar			
	0 (ref)		
	1	0.1489	0.77 (0.54, 1.10)
	2	0.4704	0.85 (0.54, 1.33)
	3	0.2482	0.73 (0.42, 1.25)
	4	0.2546	0.73 (0.42, 1.26)
	5	0.1263	1.30 (0.93, 1.81)

Note. * indicates significance at p < 0.05. Models were adjusted for age, sex, race, body mass index category, and income group. Reference group for dependent variable = zero days using cigarettes.

**Table 6:** An ordinal logistic regression analysis of the association between beverage consumption and number of days using an e-cigarette in the last 30 days among grade 9 to 12 students classified as e-cigarette users in 2016/2017 of the COMPASS study.

	Number of days consuming	P-value	OR (CI)
Days per school week drinking sugar-sweetened beverages			
	0 (ref)		
	1	0.3658	1.08 (0.91, 1.28)
	2	0.0950	1.17 (0.97, 1.41)
	3	0.6691	1.04 (0.87, 1.24)
	4	0.3380	1.13 (0.88, 1.44)
	5	< 0.0001*	1.65 (1.31, 2.07)
Days per school week drinking high energy drinks			
	0 (ref)		
	1	< 0.0001*	1.47 (1.25, 1.73)
	2	< 0.0001*	1.80 (1.42, 2.29)
	3	0.0024*	1.75 (1.22, 2.50)
	4	0.0251*	1.55 (1.06, 2.27)
	5	< 0.0001*	3.45 (2.32, 5.12)
Days per school week drinking coffee/tea with sugar			
	0 (ref)		
	1	0.8673	1.01 (0.86, 1.19)
	2	0.0109	1.23 (1.05, 1.45)
	3	0.0008*	1.42 (1.16, 1.73)
	4	0.0033*	1.43 (1.13, 1.81)
	5	< 0.0001*	1.62 (1.36, 1.93)
Days per school week drinking coffee/tea without sugar			
	0 (ref)		
	1	0.6840	1.05 (0.84, 1.31)
	2	0.8938	1.02 (0.77, 1.34)
	3	0.9642	1.01 (0.73, 1.40)
	4	0.7263	1.07 (0.75, 1.52)
	5	0.0584	1.24 (0.99, 1.55)

Note. * indicates significance at p < 0.05. Models were adjusted for age, sex, race, body mass index category, and income group. Reference group for dependent variable = non e-cigarette users.

## 4 Discussion

Our study demonstrated a consistent and significant association between beverage consumption and smoking and e-cigarette behaviours within the COMPASS cohort. In line with our hypotheses, the greater the consumption of SSBs, high energy drinks or coffee/tea, the greater likelihood of being a current smoker, a former smoker, currently using an e-cigarette, smoking more cigarettes each month and using an e-cigarette more each month after controlling for age, sex, BMI, school median income, and ethnicity. We found that e-cigarette use and cigarette smoking demonstrate similar associations across different types of caffeinated beverage consumption and the most robust association existed between high energy drink consumption and smoking behaviours. These findings suggest a need for future research examining temporal associations between the two behaviours.

In Canada, tobacco is the leading risk factor contributing to morbidity and mortality (Alam et al., 2019) and the use of e-cigarettes is on the rise in youth and young adults (Levy et al., 2018), suggesting a need to investigate moderators of smoking behaviours in youth. Caffeine consumption has been associated with smoking behaviour in adults (Swanson et al., 1994, Istvan and Matarazzo, 1984, Klesges et al., 1994). Caffeine is consumed in a variety of ways beyond coffee, especially by adolescents, with SSB representing a large source of caffeine (Branum et al., 2014). In the current study we aimed to investigate the association between caffeinate beverage consumption and smoking behaviour. In all models, the more frequently SSBs were consumed, the association with smoking behaviour strengthened, which aligns with current literature in adults. Kearns and colleagues (Kearns et al., 2018) demonstrated a significant increase in nicotine consumption with increased SSB consumption in a young adult population (mean age: ∼24 years), and in a large American cohort (n = 12,214), SSB consumption predicted smoking behaviour in adults (Age: >18 years) (Kristal et al., 2015). Further, high energy drinks, a sub-class of SSB, are a large source of caffeine in youth (Branum et al., 2014). Here we identified high energy drink consumption to be the strongest predictor of smoking behaviour, though these beverages contain varying caffeine and sugar amounts (Kaminer, 2010). High energy drink consumption is associated with several negative health behaviours in adolescents, such as smoking cannabis and smoking (Miller, 2008, Azagba et al., 2014). High energy drinks have predicted e-cigarette use and smoking in Finnish adolescents as well (Kinnunen et al., 2018). This may be related to altered taste perceptions in smokers, causing overconsumption of sweeteners (Kearns et al., 2018, Pepino and Mennella, 2007, Sato et al., 2002).

SSBs and caffeine-rich beverages are easily accessible to adolescents and potentially influence smoking/vaping behaviour through both physiological and psychological mechanisms (Benowitz et al., 1989, Colby et al., 2017, Fredholm et al., 1999, Hair et al., 2009, McRobbie and Hajek, 2004). Physiological mechanism have been identified in animal models, in which chronic exposure to caffeine potentiates nicotine self-administration (Shoaib et al., 1999, Prada and Goldberg, 1985, Yasar et al., 1997). Caffeine exposure accelerates the development of nicotine self-administration, suggesting a cumulative stimulation of the dopaminergic pathway via both adenosine receptor antagonization (caffeine) and cholinergic stimulation (nicotine) (Treur et al., 2016). Genetic factors have been shown to explain part of the association between smoking and caffeine consumption, in that increased cigarette smoking may causally increase caffeine consumption (Bjørngaard et al., 2017, Marczinski, 2011). Psychological mechanisms include the identification of smoking and e-cigarette use as “risky behaviours”. Additionally, the use of high energy drinks and caffeine are also considered “risky behaviours” (Colby et al., 2017, Hair et al., 2009). Youth engaging in any one health-compromising behaviour might engage in many health-compromising behaviours (Owens et al., 2014). High energy drink consumption was associated with increased sexual risk-taking, fighting, and marijuana use in undergraduate students (Miller, 2008) and risky behaviours such as smoking, taking drugs, drinking, and sexual behaviour have been shown to co-occur in late-adolescence (Hair et al., 2009). Moreover, in adults and youth alike, it is understood that stress, anxiety and depression are related to SSBs and smoking behaviours (Richards and Smith, 2016, Fluharty et al., 2017, Chaiton et al., 2009, Iwamoto and Smiler, 2013). Finally, in youth, social factors (i.e., peer pressure) have been related to substance use (Godin et al., 2018).

### 4.1 Limitations

This is the first study to identify an association between a wide range of beverage consumption and smoking behaviour in Canadian youth and identify high energy drink consumption as the strongest correlate of smoking behaviour; however, there are several limitations to consider. First, the COMPASS study relies on self-report measures that are subject to biases, such as social desirability and response bias, which may lead to under-reporting of both beverage consumption and smoking behaviour. Second, the analysis did not control for academic achievement, as the COMPASS study does not link academic performance records to the participants but rather uses two self reported measures of math and english academic achievement. The analysis was run including the available measures of academic achievement (not reported in this manuscript) and did not alter the findings. Additionally, the COMPASS study uses a convenience sampling strategy and, therefore, may not be representative of all adolescents in Canada. The use of cross-sectional study designs does not allow for causation to be determined, and therefore, we are unable to comment if beverage consumption is causing smoking behaviour or vice-versa. Finally, the beverage measures in the COMPASS study are not validated, which may result in some categories of SSBs, such as flavoured milk, to be excluded. The measures also do not differentiate between caffeinated and non-caffeinated SSB. Additionally, artificially sweetened ‘diet’ beverages can also contain caffeine and are not included in the analysis.

## 5 Conclusions

Despite these limitations, this exploratory work suggests an association between the consumption of SSB, high energy drinks and coffee/tea and increased likelihood of being a current smoker or e-cigarette user. Additionally, SSB, high energy drinks and coffee/tea consumption is associated with the frequency of smoking/using an e-cigarette per month among adolescents. This study was the first to compare the association of multiple types of beverages (i.e., high energy drink, SSBs, and coffee/tea) between both combustible cigarettes and e-cigarettes. Future work should examine the directionality of this association and explore further the potential mechanisms contributing to this association.

## 6 Implications and contribution

Given the health consequences of smoking and e-cigarette use and excessive SSB consumption, policy initiatives to prevent smoking initiation and restrict access to SSB and especially high energy drinks, for example, through taxes on sugar-sweetened beverages (Faulkner et al., 2011), need ongoing attention and implementation, as they may have synergistic effects on both health behaviours. Additional educational interventions addressing the health risks of these behaviours and their interaction may also be needed to prevent future use.

## 7 Doi

The COMPASS study has been supported by a bridge grant from the CIHR Institute of Nutrition, Metabolism and Diabetes (INMD) through the “Obesity – Interventions to Prevent or Treat” priority funding awards (OOP-110788; awarded to SL), an operating grant from the CIHR Institute of Population and Public Health (IPPH) (MOP-114875; awarded to SL), a CIHR project grant (PJT-148562; awarded to SL), a CIHR bridge grant (PJT-149092; awarded to KP/SL), a CIHR project grant (PJT-159693; awarded to KP), and by a research funding arrangement with Health Canada (#1617-HQ-000012; contract awarded to SL).

## CRediT authorship contribution statement

**Matthew James Fagan:** Conceptulization, Investigation, Methodology, Visualization, Writing-original draft, Writing-review & editing. **Katie Mary Di Sebastiano:** Conceptulization, Investigation, Methodology, Visualization, Writing-original draft, Writing-review & editing. **Wei Qian:** Data curation, formal analysis, Writing-review & editing. **Scott Leatherdale:** Funding aquisition, Methodology, Resources, Writing-Review & editing. **Guy Faulkner:** Conceptulization, Investigation, Methodology, Visualization, Writing-original draft, Writing-review & editing, Supervison.

## Declaration of Competing Interest

The authors declare that they have no known competing financial interests or personal relationships that could have appeared to influence the work reported in this paper.
